# The Adsorption Behavior of Surfactants on Hydrophobic Surfaces: Dissipative Particle Dynamics Study

**DOI:** 10.1021/acs.jpcb.6c01452

**Published:** 2026-06-23

**Authors:** Tomasz Staszewski, Małgorzata Borówko

**Affiliations:** Department of Theoretical Chemistry, Institute of Chemical Sciences, Faculty of Chemistry, Maria Curie-Skłodowska University in Lublin, Lublin 20-031, Poland

## Abstract

We study the behavior of a model surfactant that mimics sodium dodecyl sulfate (SDS) near a hydrophobic surface. We focus on the surfactant adsorption over a wide range of surfactant concentrations. The obtained adsorption isotherm is consistent with the experimental results. We explore the mechanism of surfactant adsorption on the solid surface and the interplay between the adsorption of surfactants and their aggregation both on the substrate and in the bulk solution. The self-assembly of surfactant molecules is analyzed in detail. We found that adsorption of whole micelles (rather than single molecules) from the solution is the dominant mechanism for surfactant accumulation on surfaces.

## Graphical abstract

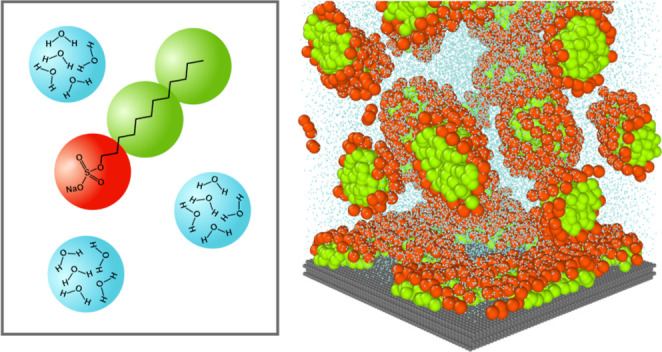

## 1 Introduction

Adsorption of surfactants from solutions onto solid surfaces plays a significant role in industrial processes involving wetting, adhesion, detergency, the stabilization of colloidal dispersions, enhanced oil recovery, and many others. The physicochemical properties of surfactants were concisely described in several monographs.^1–5^ The behavior of surfactants on various substrates was a subject of extensive experimental and theoretical research.^5^ The studies used models of various degrees of complexity, from full-atomistic, through coarse-grained, to simple phenomenological ones. In the recent two decades, molecular simulations have become one of the most important research tools that enable us to understand the interfacial properties of surfactants on the microscopic level.^5^ These methods were used to study the behavior of surfactants on different substrates, including flat homogeneous^6–11^ and heterogeneous^12–16^ surfaces, cylindrical pores,^17–21^ nanotubes^22–24^ and polymer brushes.^25,26^ Sufactants near flat surfaces were a subject of numerous molecular simulations that focused on the interplay between their aggregation and adsorption. Lisal et al.^10^ studied the micellization of nonionic surfactants in bulk aqueous solutions, and their adsorption and self-organization on a planar hydrophobic surface by dissipative particle dynamics (DPD). They found that surfactants strongly adsorb on the hydrophobic surface, adopting lying-down configurations and forming hemispheres which are in equilibrium with the spherical micelles in the bulk. All-atomic MD simulations were conducted^11^ for anionic and nonionic surfactants adsorbed on silica substrates with a varying degree of hydroxylation. The aggregate morphology was discussed and compared to available experimental data. In a comprehensive review article, Striolo and Grady^12^ summarized the results on surfactant adsorption on nanostructured surfaces characterized by surface roughness and chemical heterogeneity. The effect of confinement on the self-assembly of surfactants was also studied via molecular simulations. Arai et al.^17^ reported the results of DPD simulations of the self-assembly of surfactants inside nanotubes with hydrophilic, hydroneutral, and hydrophobic walls. They analyzed the effect of the nature of walls on the system morphologies and polymorphic transitions. Adsorption of nonionic surfactants at the surface of mesoporous silica glass was studied by a combination of adsorption measurements and DPD simulations.^18–20^ The admicelle formation at the pore walls was found. Moreover, Wu et al.^21^ investigated experimentally and through all-atom molecular dynamics simulations (MD) the self-assembly of a model nonionic surfactant triethylene glycol monohexyl ether in tubular nanopores of the SBA-15 silica material. Moreover, adsorption of surfactants on carbon nanotubes was studied via molecular dynamics.^22,23^ They discussed parameters that affected adsorption and morphology of surfactant aggregates on the nanotubes. The extensive all-atom molecular dynamics simulations were also carried out to study the morphology of sodium dodecyl sulfate (SDS) surfactant aggregates adsorbed on single-walled carbon nanotubes.^24^ The coarse-grained molecular dynamics simulations were used to investigate the behavior of amphiphilic molecules on the polymer brushes.^25,26^

The adsorption of surfactants on liquid/solid interfaces has also been investigated using other theoretical approaches. One of the first theoretical treatments was that proposed by Zhu and Gu.^27^ They assumed a two-step mechanism of adsorption. First, a single surfactant molecule was adsorbed through interactions with the surface. Then, successive surfactants attached to it due to hydrophobic interactions, forming hemicelles. Based on this adsorption mechanism, they derived a simple analytical equation for the adsorption isotherm that can interpret various types of surfactant isotherms reported in the literature, including Langmuir-type, S-type, and two-plateau-type isotherms.^3,28^

Much later, more advanced density functional theory was employed to study the behavior of surfactants on solid surfaces. This method was used to study the competition between the aggregation of amphiphilic molecules and their adsorption at different substrates^29^ and the behavior of amphiphilic particles on surfaces covered by the polymer-tethered brushes.^30^ Although much experimental and theoretical work has been done in this field, the mechanism of surfactant adsorption on solids still requires further investigation.

In this work, we employed the DPD simulation to study the behavior of a model surfactant that mimics sodium dodecyl sulfate (SDS) near a hydrophobic surface. We focused on the SDS adsorption over a wide range of surfactant concentrations. Our purpose was to explore the mechanism of surfactant adsorption on the solid surface and the interplay between the adsorption of surfactants and their aggregation both on the substrate and in the bulk solution.

The remainder of the paper is organized as follows. The DPD method, coarse-grained models, and simulation protocol are outlined in Section 2. Our results are presented and discussed in Section 3. Finally, the conclusions are summarized in Section 4.

## 2 Simulation Models and Methods

### 2.1 Dissipative Particle Dynamics

Molecular simulation has become a complementary tool to experiments to investigate the self-assembly and adsorption of surfactants at surfaces. It allows us to tune the surfactant-surface and surfactant–surfactant interactions through the choice of properties of surfactants, solvents, and solid surfaces. The time and length scales for surfactant aggregation and adsorption on a solid surface are too long to be conveniently studied by all-atom molecular dynamics.^10^ Therefore, coarse-grained molecular dynamics or dissipative particle dynamics (DPD) are often used.^31–33^ In these methods, molecules or their fragments are grouped into mesoscopic beads, which significantly speeds up calculations. The dissipative particle dynamics model consists of particles that correspond to coarse-grained beads representing clusters of molecular structures rather than individual atoms. The beads interact with each other through the force that can be broken into the following parts^31–33^

1 $$F_{i}=\sum_{j\neq i}\left(F_{ij}^{C}+F_{ij}^{D}+F_{ij}^{R}+F_{ij}^{B}\right)$$

where the C, D, R, and B stand for conservative, dissipative, random, and bonding interactions. The forces exerted on particle *i* by particle *j* are

2 $$F_{ij}^{C}=\{\begin{array}{ll} a_{ij}\left(1-r_{ij}/r_{c}\right)){\mathrm{r̂}}_{ij} & r_{ij}<r_{c}\\ 0 & r_{ij}\geq r_{c} \end{array}$$

3 $$F_{ij}^{D}=-\gamma \omega^{D}\left(r_{ij}\right)\left({\mathrm{r̂}}_{ij}\cdot v_{ij}\right){\mathrm{r̂}}_{ij}$$

4 $$F_{ij}^{R}=\sigma \omega^{R}\left(r_{ij}\right)\xi_{ij}\Delta t^{-1/2}{\mathrm{r̂}}_{ij}$$

5 $$F_{ij}^{B}=-k_{s}\left(r_{ij}-r_{0}\right){\mathrm{r̂}}_{ij}$$

In the above, the vectors **r**_*ij*_ = **r**_*i*_ – **r**_*j*_ and **v**_*i*_ = **v**_*ij*_ – **v**_*j*_ characterize the distance between particles *i* and *j* and their relative velocity, respectively, *r*_*ij*_ = |**r**_*ij*_|. The ${\mathrm{r̂}}_{ij}$ is the unit vector in the direction of **r**_*ij*_. The pair DPD interaction parameter *a*_*ij*_ determines the strength of the conservative forces and describes the interactions between particles *i* and *j*. However, the *r*_*c*_ is the cutoff distance that defines the effective interaction range, and it is the length unit in the DPD simulation. The variation of the friction coefficient and random force with distance is represented by the weight functions ω^D^ and ω^R^, respectively. The parameter ξ_*ij*_ is a random number selected following a Gaussian distribution with zero mean and unit variance, the γ is a coefficient controlling the strength of the frictional forces between the DPD beads, and the σ determines the magnitude of the random pair force between particles. The bonds between beads are modeled by spring forces with spring constant *k*_s_ and the equilibrium bond length, *r*_0_. The requirement of the canonical distribution sets two conditions on the weight functions and the amplitudes of the dissipative and random forces^34^

6 $$\omega^{D}={\left[\omega^{R}\left(r_{ij}\right)\right]}^{2}$$

7 $$\sigma^{2}=2k_{B}T\gamma$$

### 2.2 Coarse-Grained Models

We consider the adsorption of surfactants on a hydrophobic surface. We use the model introduced by Suttipong et al.^13^ to study aqueous solutions of sodium dodecyl sulfate (SDS) near a planar surface. In this model, one DPD water bead (*W*) represents five water molecules, and surfactant is modeled by three DPD beads: one of them mimics the headgroup (*H*), and two correspond to the tail (*T*). All beads have the same diameters. The reduced density, defined as the number of beads in a cubic volume of radius *r*_*c*_, is ρ_DPD_ = 5 beads per *r*_*c*_^3^. This means that *r*_*c*_ = 9.0856 Å. The surfactant beads are connected by harmonic bonds to form a single chain. The bond length *r*_0_ = 10.4 Å and the spring constant *k*_s_ = 100*k*_B_ *T*/*r*_*c*_.^13,34^

The solid surface is built with beads treated as rigid bodies organized in a square lattice with nearest-neighbor distance 0.35*r*_*c*_ in *x* and *y* directions. Four planes of solid beads (S) are stacked on top of each other with an interlayer distance 0.35*r*_*c*_. Two consecutive planes are shifted with respect to each other by the shift distance 0.175*r*_*c*_ along both *x* and *y* directions.^13^ The wall can mimic carbon-type surface. The DPD parameters *a*_*ij*_ are calculated according to the Groot and Warren method.^32^ The parameter for water–water interaction was chosen based on the water compressibility at normal conditions, *a*_*ii*_ = 75*k*_B_ *T*/ρ_DPD_. Under assumed density and temperature, *a*_*ii*_ = 15. The water–surfactant parameters were taken from the article by Kuo et al.^35^ They determined the averaged pair interaction energies using Monte Carlo simulations and calculated the Flory–Huggins parameter χ_*ij*_. Then, the χ_*ij*_ was used to determine the maximum repulsion *a*_*ij*_ between the particle pair *i* – *j* at given particle density, *a*_*ij*_ = *a*_*ii*_ + 1.451χ_*ij*_. Notice, however, that in our simulations the surfactant is composed of one head and two tail beads,^13^ while Kuo et al.^35^ represented SDS molecule with only one head and one tail bead.

The DPD parameters *a*_*ij*_ were validated by comparison with the critical micelization concentrations estimated from bulk simulations and experiments.^13,34,36–41^

The interactions between the surface beads and the surfactant tail beads were set to replicate tail–tail interactions, while head-surface interactions replicated tail–head interactions. All interaction parameters implemented in this work are collected in Table 1.

**Table 1:** Repulsion Parameters *a*_*ij*_, for Water (*W*), Surfactant Headgroup (*H*), Surfactant Tail (*T*), and Hydrophobic Surface (*S*) Beads

	*W*	*H*	*T*	*S*
*W*	15	0	81	81
*H*		15	69	69
*T*			15	15
*S*				15

In this model, electrostatic interactions are not explicitly taken into consideration. Therefore, the model cannot reproduce all physical properties observed for ionic surfactants.^13^

### 2.3 Simulation Protocol

Simulations were carried out using the simulation package LAMMPS,^42,43^ with the DPD force fields based on pairwise interactions, and the reduced temperature *k*_B_ *T* = 1. The random and dissipative parameters were set to σ = 3 and γ = 4.5.^32^ The time step Δ*t* = 0.03τ was used to integrate the equations of motion. The simulation time scale is τ = 5.2 ps.

A rectangular box of dimensions *L*_*x*_, *L*_*y*_, *L*_*z*_, where *L*_*x*_ = *L*_*y*_ = 21*r*_*c*_ and *L*_*z*_ = 42*r*_*c*_ was used with periodic boundary conditions along *x* and *y* directions. The surfaces are located at *z* = 0 and *z* = 42*r*_*c*_. The distance between the surfaces was large enough to allow the existence of the bulk solution in the central part of the system. We treated *r*_*c*_ as the length unity and in the rest of the work we use the reduced z-coordinate, z* = *z*/*r*_*c*_.

To determine the adsorption isotherm, we performed a series of independent simulations for systems differing in surfactant concentration. In each case, we performed several simulations starting from different initial configurations. This procedure corresponds to the experimental method of determining adsorption isotherms, in which the adsorbent is flooded with a solution of a specific concentration and, after establishing equilibrium, the concentration in the bulk phase is measured, and the adsorption excess is calculated. Simulations allow us to directly determine the number of molecules adsorbed on the surface.

Equilibration runs lasted at least 10^7^ DPD streps. The average values were calculated from following 10^7^ configurations. Simulations were performed for different total numbers of surfactant molecules present in the system, varying them from *N* = 10 to *N* = 5000. This corresponds to molar concentrations ranging from 1.28 mM to 640.4 mM.

During simulations, we determined the local densities for surfactant beads (segments) and water, ρ_sα_ (α = *H*, *T*, *W*). The density profiles were calculated as averages of those obtained for both walls. The total density of surfactant segments is the following sum, ρ_s_ = ρ_sH_ + ρ_sT_. We also calculated the number density of surfactant molecules, ρ = ρ_s_/3. We determined the value of this density in the bulk part of the system, ρ_b_. Then, we estimated the real adsorption of surfactants, defined as the number of surfactant molecules touching the surface (so-called primary adsorption). A molecule was classified as adsorbed when at least one of its segments was at a distance z* ≤ 3.5 or z* ≥ 38.5. An effective instrument to gain insight into the system morphology is cluster analysis. We analyzed the aggregation of surfactant molecules in the inhomogeneous systems. Two molecules were classified as belonging to an aggregate when their distance is smaller than *r*_*c*_. The number of surfactant molecules in a given aggregate defines the cluster size. We estimated cluster-size distributions *P*(*s*), average cluster sizes, ⟨*s*⟩, and average numbers of clusters in the bulk solution and on the surface, ⟨*N*_cl_⟩.

The OVITO software was used for visualization of the equilibrium configurations.^44^

## 3 Results and Discussion

We performed simulations of aqueous SDS solutions at a model hydrophobic surface, varying the surfactant concentration in a wide range. First, we dealt with the behavior of the surfactants in selected bulk systems. In all cases, we observed the formation of micelles. Then, we studied the surfactant–water solutions in systems involving solid surfaces. We focus on the influence of surfactant concentration on adsorption and micellization.

We start with the analysis of the behavior of the studied systems at equilibrium. In Figure 1, we show the determined adsorption isotherms. The adsorption is expressed as the number of adsorbed surfactant molecules per unit surface, *N*_s_, and plotted as a function of the bulk surfactant density (a) and in relation to the total number of surfactant molecules (b). For low surfactant concentrations, the adsorption increases rapidly (a). Between ρ_b_ = 0.059 and ρ_b_ = 0.122, a flattening in the adsorption is observed. Further increase in concentration causes a slight jump in adsorption. At high bulk concentrations, the adsorption gradually reaches its saturation value. The adsorption is a result of a complex interplay between interactions in the bulk solution, leading to the micellization and interactions with the surface associated with the formation of admicelles. This issue is discussed in detail below. The adsorption plotted as a function of the total number of surfactant has a similar shape (b). In particular, the inflection point is clearly visible at *N* = 2000.

**Figure 1:**
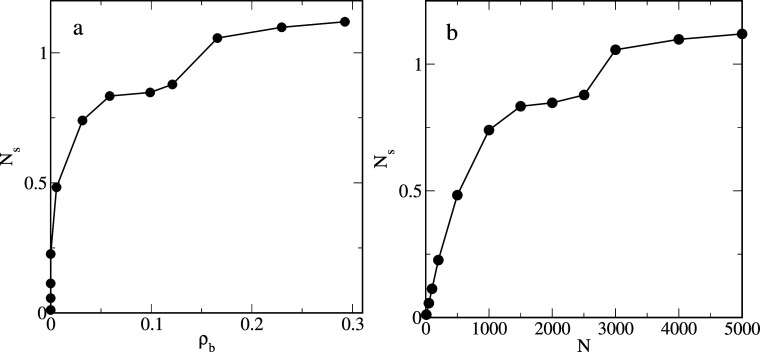
Adsorption isotherms. The number of adsorbed surfactant molecules per unit surface, *N*_s_, is plotted as a function of the bulk number of surfactant density (a) and in relation to the total number of surfactant molecules (b). The circles correspond to simulation points.

The obtained isotherm shows the obvious difference compared to the Langmuir adsorption isotherm^3^ for monolayer adsorption process with negligible lateral interaction between adsorbed molecules. The Langmuir type of adsorption isotherm features a monotonous increase in the magnitude of adsorption according to bulk concentration without any inflection points. However, the simulation isotherm has an inflection point, suggesting a structural transformation in the surface layer.

The isotherm presented in Figure 1a is similar to the isotherm of adsorption of SDS on polystyrene latex^28^ that had been interpreted in the framework of Zhu-Gue theory.^27^ The authors suggested that the process was dominated by the formation of admicelles on the surface (the fitted value of the micellization constant was much greater than the adsorption constant). The strong aggregation of surfactants at the hydrophobic surfaces was confirmed by atomic force microscopy investigations.^45^

The changes in adsorption magnitude can be analyzed by plotting the fraction of the adsorbed molecules, *N*_s_/*N*, as a function of the total number of surfactants in the system (Figure 2). If the number of surfactants introduced to the system is very small, all molecules adsorb onto the surfaces. After exceeding a certain threshold value, a part of the surfactants remains in the bulk part of the system. In the case *N* = 500, most of the surfactants (about 85% of SDS molecules) were adsorbed. As *N* increases, the fraction of adsorbed molecules quickly decreases because the surface becomes increasingly unavailable for primary adsorption.

**Figure 2:**
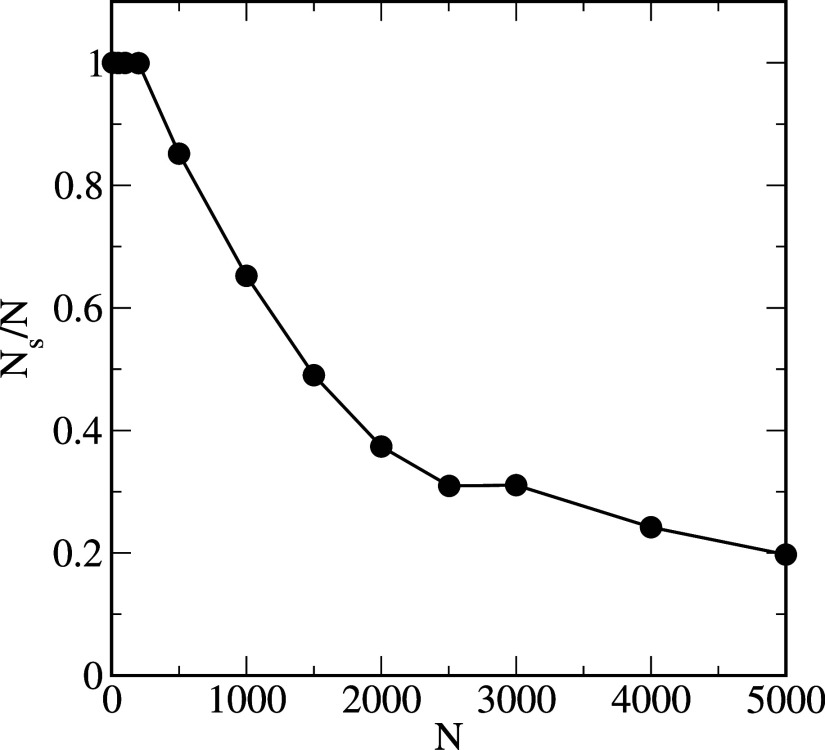
Fraction of the adsorbed surfactant molecules *N*_s_/*N*, as a function of the total number of surfactants in the system, *N*. The circles correspond to simulation points.

It is interesting to analyze the structure of the systems at equilibrium on a molecular level. For this purpose, we calculated the density profiles of particular beads and monitored the equilibrium configurations of the systems. In Figure 3 the density profiles of hyrophobic segments, ρ_sT_(z*), hydrophilic heads, ρ_sH_(z*), and water beads, ρ_sW_(z*), are presented for selected total numbers of surfactant molecules in the system.

**Figure 3:**
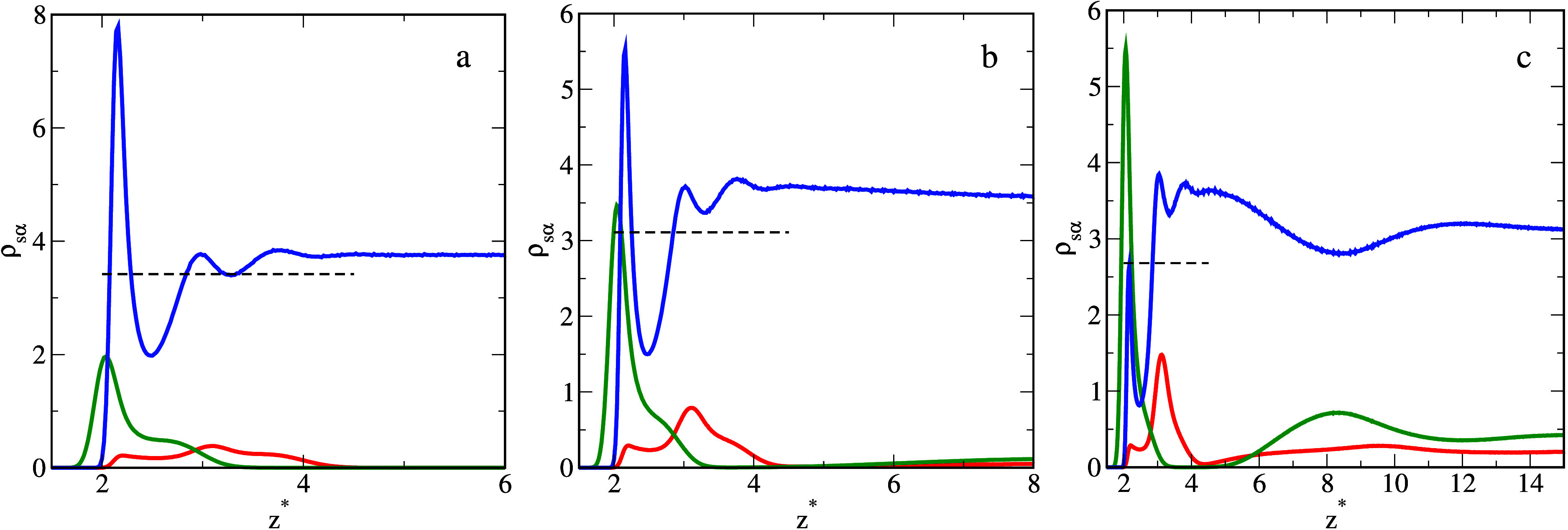
Density profiles of surfactant beads ρ_sα_(z*), where z* = *z*/*r*_*c*_, for selected total numbers of surfactant molecules in the system: *N* = 500 (a), *N* = 1500 (b), and *N* = 4000 (c). Green lines correspond to hydrophobic tail segments (α = *T*), red lines are for hydrophilic heads (α = *H*), and blue lines correspond to water beads. Dashed black lines denote the average water density in the surface layer.

The density profiles of tail segments have sharp maxima near the surface (at z* = 2.09) and for greater distances, z*, the densities, ρ_sT_, gradually decreases to minima at z* = 3.87. These well-pronounced and narrow peaks correspond to the tail segments lying immediately at the surface. In this way, the surfactants maximize the interactions of their hydrophobic tails with the surface. As the surfactant concentration in the system increases, the density profiles ρ_sT_(z*) change significantly. The first peaks in ρ_sT_(z*) become higher. In the case of dilute surfactant solutions (parts a, b), for z* > 10 the ρ_sT_(z*) smoothly approaches the bulk values. However, for the concentrated solution (part c), the second low and wide peak appears at z* = 8.27. This maximum indicates a weak secondary adsorption of surfactants. On the first adsorbed layer, successive surfactants start to accumulate. It should be emphasized here that this effect was not taken into account when determining the adsorption isotherm.

The density profiles of head segments, ρ_sH_(z*), have two peaks, the first, low maxima near the surface (at z* = 2.24) and the much higher maxima at z* = 3.14. The first peaks are slightly further from the wall than the first ρ_sT_(z*) peaks. The density profiles ρ_sH_(z*) reflect the tendency of the hydrophilic head beads to move away from the hydrophobic surface.

In the surface layer, the average density of water (black, dashed lines in Figure 3) is lower than its bulk density due to surface hydrophobicity and adsorption of surfactants. Adsorption of surfactants from solutions has a competitive character,^3,20,27^ water molecules are replaced by the surfactants on the surface. On the other hand, water beads can solvate the hydrophilic heads, leading to the presence of density peaks in ρ_sW_(z*) correlated with maxima in the densities ρ_sH_(z*). The water density peaks near the surface decrease with increasing surfactant concentration. It is noteworthy that in the case of the concentrated solution (c), the water density is considerably greater above the first adsorbed layer and falls below the bulk value at a distance corresponding to the local maximum in ρ_sT_. This reflects so-called secondary adsorption.

The ρ_α_ density profiles provide information only about the changes of density with distance from the wall. They do not show how the surfactant molecules are distributed on the surface. Therefore, the analysis of the density profiles was supplemented by monitoring the snapshots. An example configuration of the system containing *N* = 500 surfactant molecules is shown in Figure 4a. In this case, there is a single spherical aggregate in the interior of the simulation box and admicelles on both surfaces. The surface clusters resemble typical hemicelles. As it is visible in the view of the surface from above (b), the isolated admicelles are chaotically distributed at the surfaces. The tail beads accumulate immediately at the surface, whereas the head beads are directed toward the bulk phase. We found that the entire micelles approached the surface and changed their structure there.

**Figure 4:**
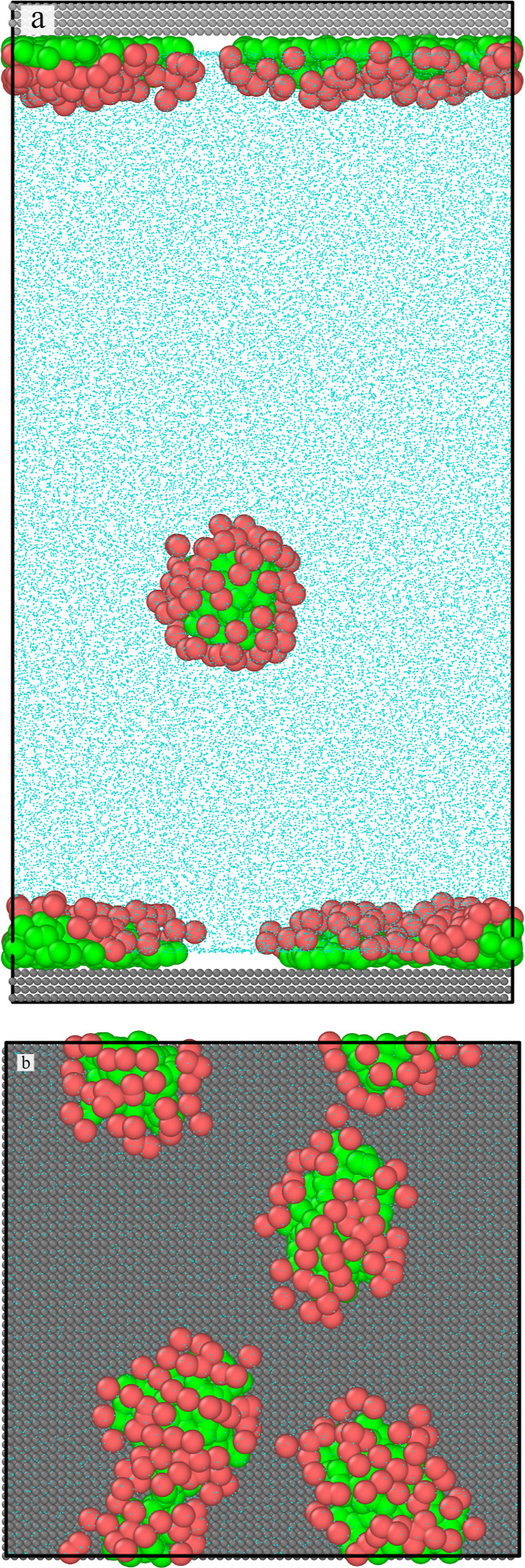
Example of equilibrium configuration of the system containing *N* = 500 surfactant molecules: (a) side view and (b) top view of the layer of adsorbed molecules. The red spheres correspond to the head beads *H*, the green spheres represent the tail beads *T*, the blue spheres are for water (*W*), and the gray spheres represent the solid beads *S*. For greater clarity of the drawing, the water molecules have been reduced.

The results obtained for *N* = 1500 are presented in Figure 5. The adsorbed surfactants are at equilibrium with a micellar solution. The micelles in the bulk phase are slightly bigger than those in a dilute solution. The admicelles join together, and larger surface aggregates are formed. In concentrated solutions (*N* = 4000), the cylindrical aggregates the cylindrical aggregates started to coexist with the spherical ones in the bulk phase, and the compact layers of adsorbed surfactants are observed on the surfaces (Figure 6). Moreover, one can see here single micelles touching the first surface layer. During secondary adsorption, entire micelles are retained, not single surfactant molecules.

**Figure 5:**
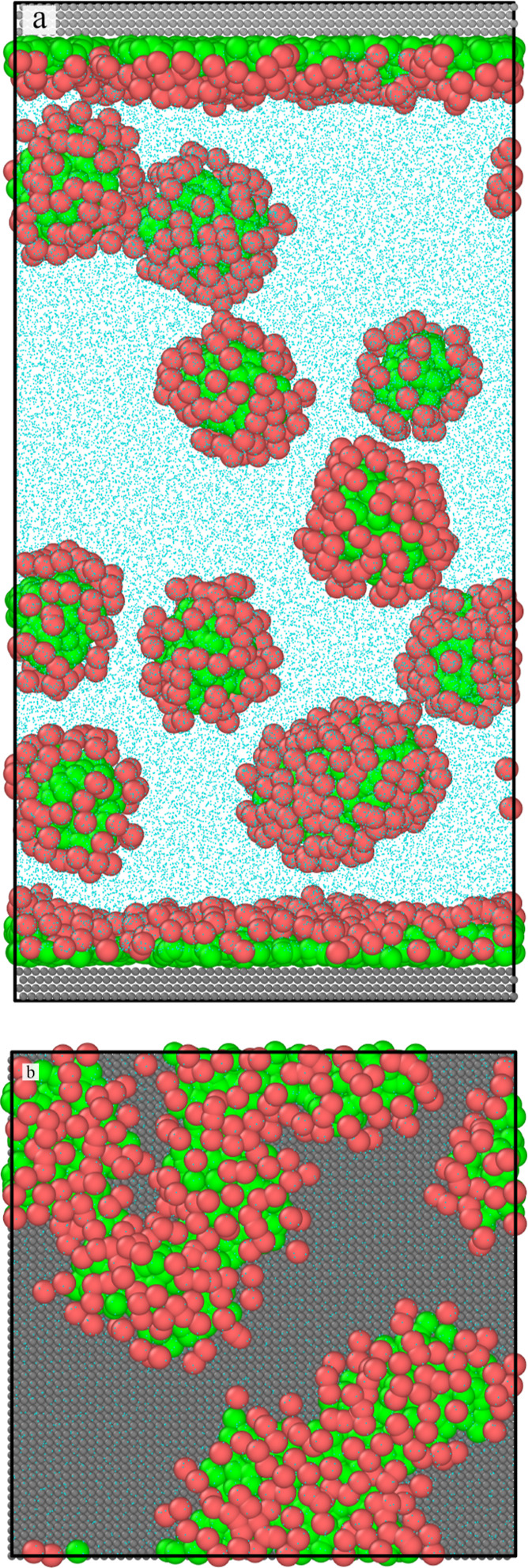
Example of equilibrium configuration of the system containing *N* = 1500 surfactant molecules: (a) side view and (b) top view of the layer of adsorbed molecules. The red spheres correspond to the head beads *H*, the green spheres represent the tail beads *T*, the blue spheres are for water (*W*), and the gray spheres represent the solid beads *S*. For greater clarity of the drawing, the water molecules have been reduced.

**Figure 6:**
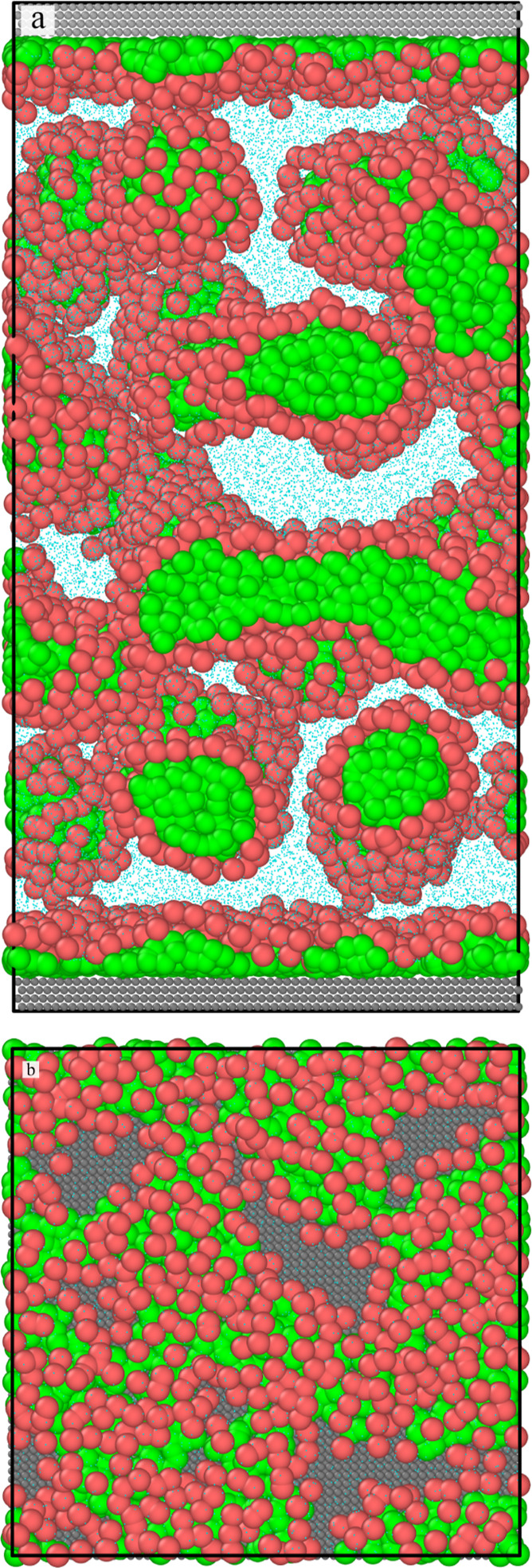
Example of equilibrium configuration of the system containing *N* = 4000 surfactant molecules: (a) side view and (b) top view of the layer of adsorbed molecules. The red spheres correspond to the head beads *H*, the green spheres represent the tail beads *T*, the blue spheres are for water (*W*), and the gray spheres represent the solid beads *S*. For greater clarity of the drawing, the water molecules have been reduced.

In Figure 7, the distributions of cluster (micelle) sizes in the bulk (a) and the surface-influenced (b) parts of the system are presented. The most probable cluster sizes are marked by the vertical dotted lines. Let us discuss the influence of surfactant concentration on their size distributions in the bulk part of the system. For *N* = 500 (black points), the sharp maximum at *s* = 67 is observed. As it is visible in the corresponding snapshot (Figure 4), we found only one micelle in the system interior. Upon an increase in the total number of surfactants, the micelles get bigger. In the case of *N* = 1500, there are aggregates of size from 45 to 150 with similar probability, and a slight maximum occurs at *s* = 72. In a dense system (*N* = 4000), aggregates have sizes from 80 to 150, but there are also much larger ones, from up to *s* = 530. The most probable size is *s* = 100. The experimental micelle size (aggregation number) of SDS close to the CMC has been reported to be *s* = 55–77 molecules.^46–48^ It has also been proved that the aggregation number of micelles grows as a function of concentration.^46–48^ Moreover, extensive molecular dynamics simulations of bulk solutions of SDS^49^ showed that the aggregation number varied from 50 to 100 depending on the concentration and temperature. Thus, our simple model correctly captures the fundamental properties of the system under consideration.

**Figure 7:**
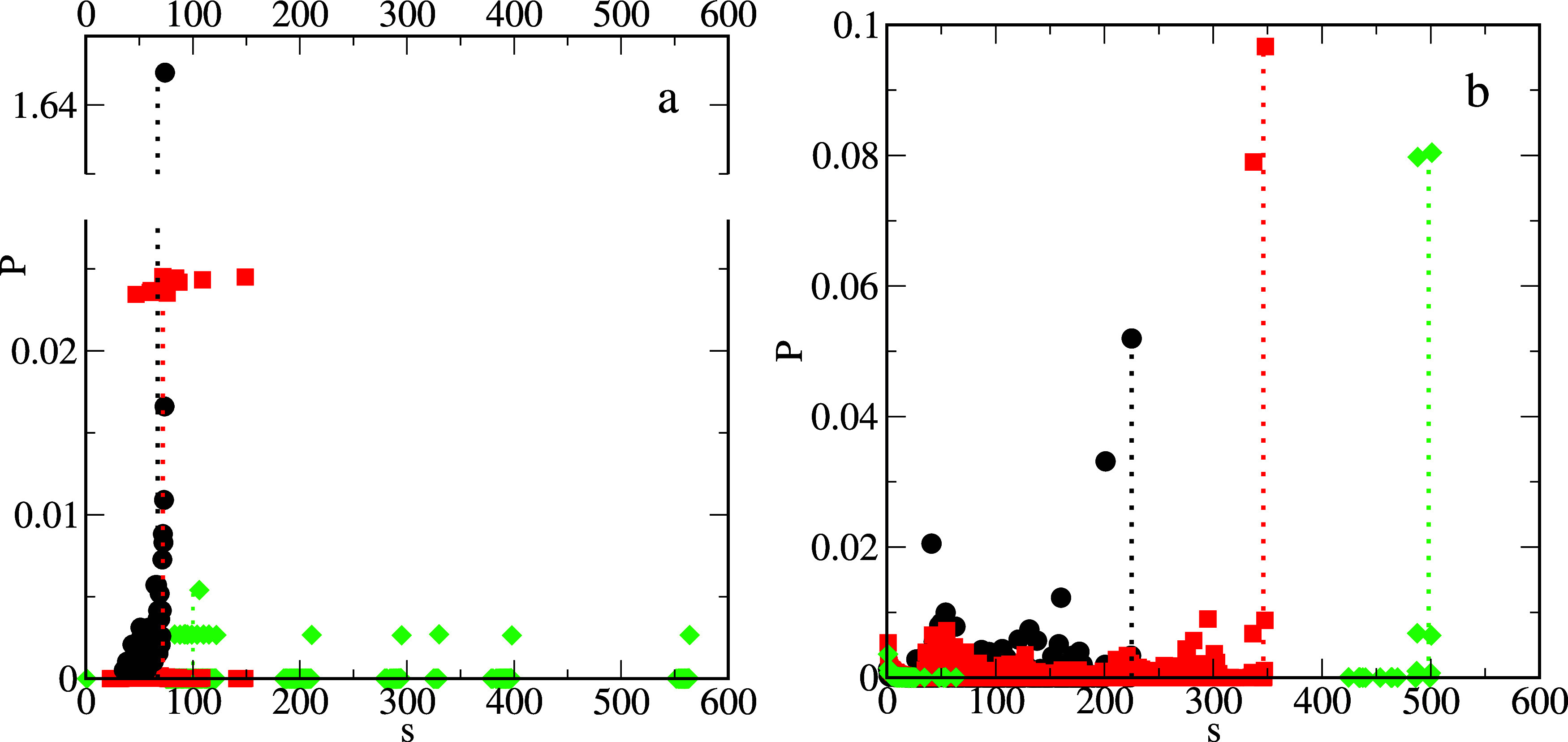
Distributions of cluster sizes, P(s), in the bulk solution (a) and on the surfaces (b).

Much larger aggregates form on surfaces. For *N* = 500, their sizes changed from 200 to 300 with a sharp maximum in the distribution at *s* = 225, while for *N* = 1500, there are aggregates of size from 340 to 350 and a high maximum appears at *s* = 346. If *N* = 4000, then 80 < *s* < 500, however, the most likely scenario is the formation of very large clusters containing *s* = 498 surfactant molecules.

For all considered systems, we have determined the average cluster sizes ⟨*s*⟩ in the bulk and surface phases. In Figure 8a, we plotted the average cluster sizes as a function of the total number of surfactants. The average cluster sizes increase with increasing surfactant concentration. The size of micelles in the bulk solution slowly increases, but for *N* > 3000, the changes become quicker. In the case of the surface clusters, this increase is considerably slower when *N* > 1000 and inflection point in the function ⟨*s*⟩ vs *N* is visible (compare the isotherm in Figure 1b). Further increase in *N* causes a gradual increase in the average size, until a plateau is reached (*N* > 3000).

**Figure 8:**
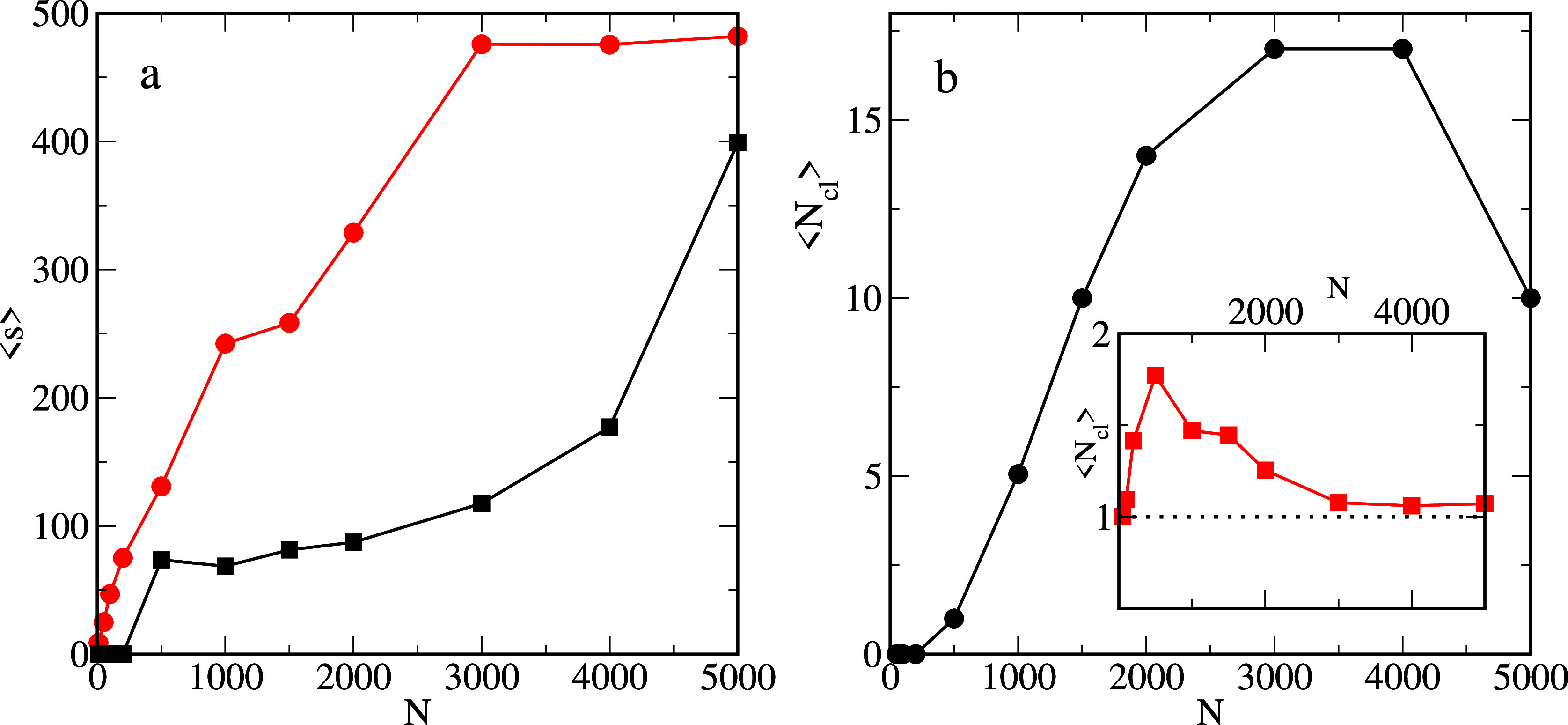
Average cluster size (a) and the average number of clusters (b) in the bulk solution (black lines) and on the surfaces (red lines) as functions of the total number of surfactant molecules in the system. The symbols correspond to simulation points.

Figure 8b shows the number of clusters plotted as a function of the total number of surfactants. In the bulk part of the system, the number of micelles increases, it achieves a maximum, and starts to decrease because the average cluster size rapidly increases for *N* > 3000. Notice that there is a point of inflection at *N* = 1500. However, the number of surface clusters increases to a low maximum at *N* = 500 and very slowly tends to unity for concentrated solutions. We monitored the equilibrium configurations of the adsorption layers (see parts (b) in Figures 4, 5, and 6). For dilute solutions, we found small clusters that were chaotically distributed on the wall. In the case of dense systems, however, we observed clusters percolating on the surface.

To explore the mechanism of adsorption, we monitored the structure of the systems during equilibration. For each system, we conducted several simulations starting from different initial configurations. In all cases, the course of the process was qualitatively the same. In initial configurations, individual surfactant molecules were randomly distributed in the aqueous medium. Then, they formed micelles and adsorbed on the surfaces. Here we present examples of the time evolution of systems for low (Figure 9) and higher (Figure 10) surfactant concentrations. In both cases, surfactants very quickly formed aggregates in the bulk phase, and at the same time, they adsorbed on the surface. When there are very few molecules in the system (Figure 9), surfactants adsorb both as single molecules and in the form of clusters. However, for a greater surfactant concentration, the micellization in the bulk solution is stronger, and adsorption of entire micelles is observed (Figure 10). The adsorbed aggregates are reconfigured into hemicelles. Then, small aggregates merge on the surface into larger ones.

**Figure 9:**
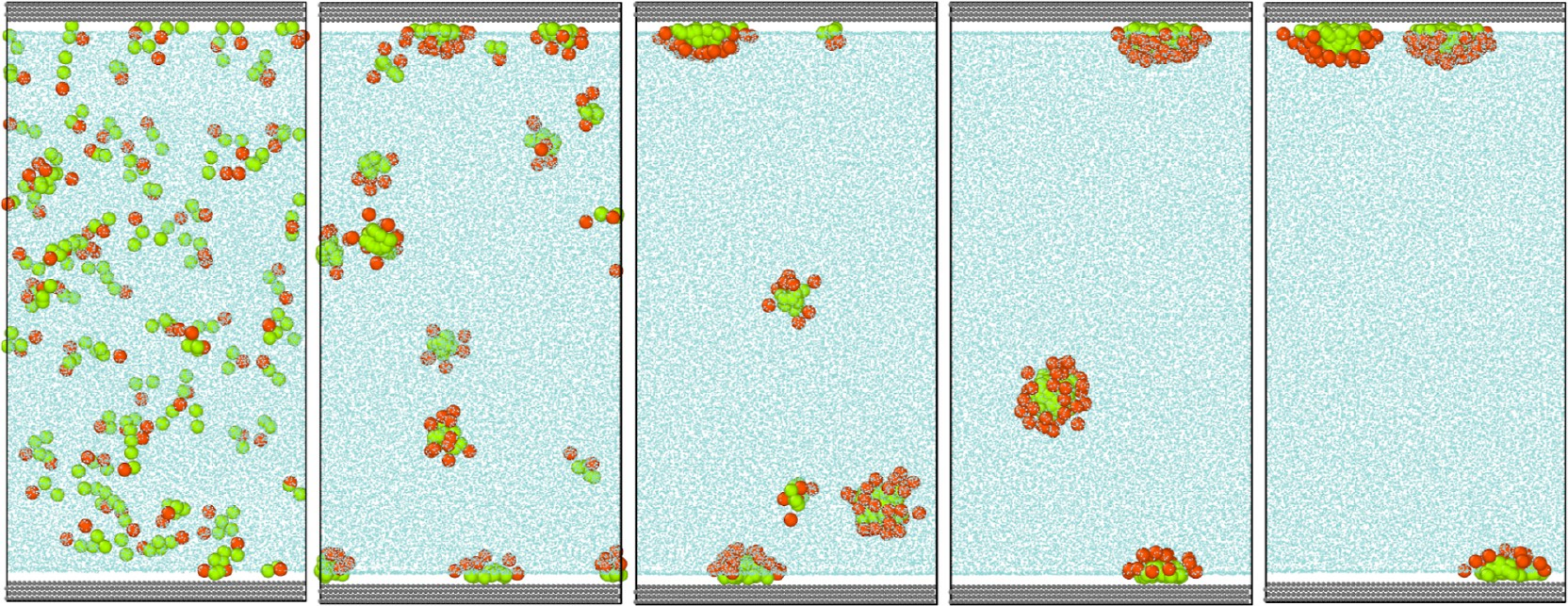
Evolution of the systems containing *N* = 100 surfactant molecules. From the left *t*: 7.8 ns, 39.0 ns, 187.2 ns, 795.6 ns. The red spheres correspond to the head beads *H*, the green spheres represent the tail beads *T*, the blue spheres are for water (*W*), and the gray spheres represent the solid beads *S*. For greater clarity of the drawing, the water molecules have been reduced.

**Figure 10:**
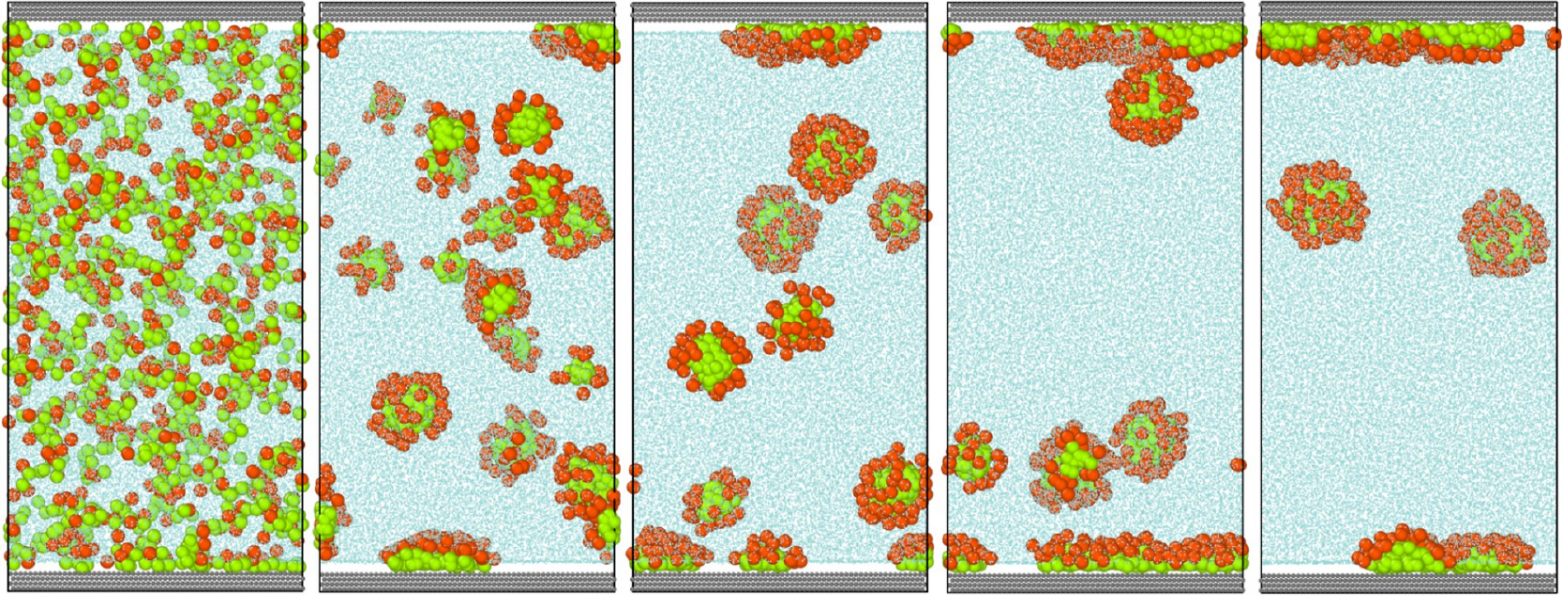
Evolution of the systems containing *N* = 500 surfactant molecules. From the left *t*: 0.0078 ns, 109.2 ns, 218.4 ns, 912.6 ns. The red spheres correspond to the head beads *H*, the green spheres represent the tail beads *T*, the blue spheres are for water (*W*), and the gray spheres represent the solid beads *S*. For greater clarity of the drawing, the water molecules have been reduced.

Thus, the simulations allowed us to learn about the adsorption mechanism at the molecular level. The adsorption mechanism observed in our simulations differs from that proposed in previous works,^27,28^ where it was assumed that micelles disintegrate in the adsorbent force field and settle on the surface as single molecules.

## 4 Conclusions

We used dissipative particle dynamics to study the adsorption behavior of surfactant molecules on a planar solid surface. The surfactant molecule mimicked sodium dodecyl sulfate. We introduced the coarse-grained model of aqueous solution of SDS in the proposed Suttipong et al.,^13^ in which one water bead represents five water molecules, and surfactant is modeled by three beads: one of them mimics the headgroup, and two correspond to the tail. The surface was represented by frozen hydrophobic beads.

First, we investigated bulk solutions of surfactants and found a strong tendency toward self-organization in all systems. Then, we systematically studied systems involving the solid surface, varying the surfactant concentration in a wide range. We focused on the interplay between aggregation in the bulk part of the system, their adsorption, and self-organization on the surface.

Here are our most important results:

1 we determined the adsorption isotherm of model surfactants that mimic SDS molecules on a hydrophobic surface in a very wide concentration range, from 2.27 mM to 442.9 mM. There are no such simulation results in the literature. The obtained adsorption isotherm had an inflection point, suggesting a structural transformation in the surface layer. A similar shape had the experimental isotherm of adsorption of SDS from aqueous solutions on polystyrene latex.^28^
2 We analyzed the morphology of the surface layers in detail. For this purpose, we calculated the density profiles of all beads and monitored the equilibrium configurations. We found that tail beads accumulated near the wall while the head beads were exposed to the bulk solution. In the surface region, a depletion in water density was observed. This reflects the competitive adsorption of surfactants on the surface. Moreover, the inclination of the particles relative to the substrate changes, which is manifested by the appearance of an inflection point in the isotherm. For sufficiently concentrated solutions, a few bulk micelles settled on top of the adsorbed layer (secondary adsorption).
3 We showed how the structure of the system changes with increasing concentration. In the bulk phase, we observed strong aggregation. At lower concentrations, micelles are spherical, for higher concentrations they coexisted with cyllindrical ones. On the surface, at lower concentrations, isolated semispherical micelles were observed; for higher concentrations, the clusters merged, and a fairly compact monolayer was formed. These findings are consistent with experimental observations.^3,50–52^

4 We performed the cluster analysis for different surfactant concentrations, we determined the cluster size distributions, and the average numbers of clusters for different total numbers of surfactants. In the bulk phase, we found that at low concentrations, the simulation aggregation numbers were in accordance with the experimental values.^46–48^ On the solid surface, we obtained higher aggregation numbers (there is no such analysis in the literature). Moreover, the number of surface clusters quickly tended to unity. This confirmed the formation of a rather compact surface layer.
5 We demonstrated the mechanism of SDS adsorption on a hydrophobic surface by analyzing the time evolution of the systems from the initial to the equilibrium configuration. We established that adsorption of whole micelles (rather than single molecules) from the solution is the dominant mechanism for surfactant accumulation on surfaces. The micelles change their shape near the walls. We found that whole micelles adsorbed at the surface and reconfigured near the wall. The micelles do not fall apart in the field of the surface as predicted by classical adsorption theories.^27,28^

6 The results of our simulations are consistent with both adsorption measurements on hydrophobic solid surfaces^28,45,50–52^ and aggregation experiments.^3,46–48^

Our simulations showed that the scenario of surfactant adsorption on solid surfaces is extremely complex. These results may be useful for optimizing technological processes based on phenomena occurring at the liquid/solid interface, such as enhanced oil recovery^53^ or removing surfactants from the environment.^54^ Furthermore, they may be helpful in the design of new surfactants directed to new, advanced applications, such as, for example, drug delivery.^55^
